# Crypt- and Mucosa-Associated Core Microbiotas in Humans and Their Alteration in Colon Cancer Patients

**DOI:** 10.1128/mBio.01315-19

**Published:** 2019-07-16

**Authors:** Azadeh Saffarian, Céline Mulet, Béatrice Regnault, Aurélien Amiot, Jeanne Tran-Van-Nhieu, Jacques Ravel, Iradj Sobhani, Philippe J. Sansonetti, Thierry Pédron

**Affiliations:** a Unité de Pathogénie Microbienne Moléculaire, Institut National de la Santé et de la Recherche Médicale (INSERM), U1202, Institut Pasteur, Paris, France; b Plate-forme de Génotypage des Eucaryotes, Pôle Biomics, Institut Pasteur, Paris, France; c EA7375 (EC2M3 Research Team), Université Paris Est Creteil (UPEC)—Val-de-Marne, Creteil, France; d Service de Gastroentérologie, APHP, Hôpital Henri Mondor, Creteil, France; e Department of Microbiology and Immunology, University of Maryland School of Medicine, Baltimore, Maryland, USA; f Institute for Genome Sciences, University of Maryland School of Medicine, Baltimore, Maryland, USA; g Chaire de Microbiologie et Maladies Infectieuses, Collège de France, Paris, France; Department of Veterinary Medicine; M2iSH, Université Clermont Auvergne; Johns Hopkins University School of Medicine; University of Chicago

**Keywords:** colon cancer, intestinal crypts, microbiota

## Abstract

We have previously identified a crypt-specific core microbiota (CSCM) in the colons of healthy laboratory mice and related wild rodents. Here, we confirm that a CSCM also exists in the human colon and appears to be altered during colon cancer. The colonic microbiota is suggested to be involved in the development of colorectal cancer (CRC). Because the microbiota identified in fecal samples from CRC patients does not directly reflect the microbiota associated with tumor tissues themselves, we sought to characterize the bacterial communities from the crypts and associated adjacent mucosal surfaces of 58 patients (tumor and normal homologous tissue) and 9 controls with normal colonoscopy results. Here, we confirm that bacteria colonize human colonic crypts in both control and CRC tissues, and using laser-microdissected tissues and 16S rRNA gene sequencing, we further show that right and left crypt- and mucosa-associated bacterial communities are significantly different. In addition to *Bacteroidetes* and *Firmicutes*, and as with murine proximal colon crypts, environmental nonfermentative *Proteobacteria* are found in human colonic crypts. *Fusobacterium* and Bacteroides fragilis are more abundant in right-side tumors, whereas Parvimonas micra is more prevalent in left-side tumors. More precisely, Fusobacterium periodonticum is more abundant in crypts from cancerous samples in the right colon than in associated nontumoral samples from adjacent areas but not in left-side colonic samples. Future analysis of the interaction between these bacteria and the crypt epithelium, particularly intestinal stem cells, will allow deciphering of their possible oncogenic potential.

## Abstract (precis)

Due to the huge number of bacteria constituting the human colon microbiota, alteration in the balance of its constitutive taxa (i.e., dysbiosis) is highly suspected of being involved in colorectal oncogenesis. Indeed, bacterial signatures in association with CRC have been described. These signatures may vary if bacteria are identified in feces or in association with tumor tissues. Here, we show that bacteria colonize human colonic crypts in tissues obtained from patients with CRC and with normal colonoscopy results. Aerobic nonfermentative *Proteobacteria* previously identified as constitutive of the crypt-specific core microbiota in murine colonic samples are similarly prevalent in human colonic crypts in combination with other anaerobic taxa. We also show that bacterial signatures characterizing the crypts of colonic tumors vary depending whether right-side or left-side tumors are analyzed.

## INTRODUCTION

Colorectal cancer (CRC) is the third most common cancer diagnosed worldwide. In 2012, CRC accounted for 1,361,000 new cases and 694,000 deaths globally (1), while in the United States alone, 95,520 new cases and 50,260 deaths were recorded in 2017 (2). The colonic microbiota may contribute to the development of colorectal cancer (3, 4). Early studies, based on culture methods, indicated an association between a few bacterial species and CRC or healthy tissues (5, 6). An increased risk for colon polyps was associated with increased abundances of *Bacteroides* and *Bifidobacteria* organisms, whereas *Lactobacillus* and Eubacterium aerofaciens were associated with the absence of tumors.

Over the past decades, the widely used 16S rRNA gene-based metataxonomics, the development of metagenomic methods based on next-generation sequencing (NGS) technology, and improved bioinformatic tools for big data analysis have afforded in-depth descriptions of the microbial compositions and functions of the gut microbiota. This has allowed for detailed comparison between feces specimens from CRC patients and those of healthy controls (7–14) and for comparison between tumor-associated microbes and microbes associated with tissues not adjacent to tumors (15–20). Among the diverse set of bacterial taxa identified in these studies, *Fusobacterium*, Bacteroides fragilis, and Parvimonas micra were found to be consistently associated with the tumor tissues (21, 22), while other taxa, like *Providencia* (23), *Roseburia*, *Ruminococcus* and *Oscillibacter* (24), and Streptococcus gallolyticus (25, 26) were found associated with tumor tissues in some, but not all, studies. Of note, one study described an association between a decreased relative abundance of *Roseburia* in fecal samples of CRC patients compared to that in healthy volunteers (27), in contrast to findings in tissue, further pointing to inconsistencies between fecal and tumor-associated microbiotas in CRC patients (28). It was also shown that microbial compositions differ between right (ascending, proximal) and left (descending, distal) cancerous colonic mucosas, with higher relative abundances of *Prevotella*, *Selenomonas*, and *Peptostreptococcus* in right colonic tumors and with higher abundances of *Fusobacterium*, *Escherichia*-*Shigella*, and *Leptotrichia* in left colonic tumors (29). In addition, right-side tumors were marked by the presence of a bacterial biofilm, unlike left-side tumors (21, 30, 31). Interestingly, *Fusobacterium* was associated with right colonic tumors in one study (32) and with both right and left colonic tumors in another (21). Moreover, it was shown that the tumor-associated microbiota could vary with the stage of the tumor (33).

Utilizing silver nitrate staining and a combination of laser-capture microdissection (LCM) and amplification of the 16S rRNA gene followed by deep sequencing, we previously showed that murine proximal colon crypts harbor a resident microbiota that we call crypt-specific core microbiota (CSCM). Regardless of the mouse line and breeding origin, this bacterial population is unexpectedly homogeneous and dominated by a restricted diversity of strictly aerobic genera, such as Acinetobacter, *Delftia*, and *Stenotrophomonas* (34). The aim of the present study was to investigate, using LCM technology and 16S rRNA gene sequencing, if human colonic crypts also harbor a consistent core microbiota and if CRC is associated with a dysbiotic core microbiota. Consequently, we characterized both the crypt-associated microbiota (CAM) and the mucosa-associated microbiota (MAM) in tumors and in their paired adjacent normal tissues in samples collected from the right and left colons of CRC patients. We compared these microbiotas with those associated with colonic biopsy specimens of healthy volunteers. Our results showed that, regardless of health status, human colonic crypts are colonized mainly with *Firmicutes* but are also colonized with Acinetobacter, *Delftia*, and *Stenotrophomonas*; however, they are in lower relative abundances than in murine proximal colon crypts. Nonfermenting *Proteobacteria* were also detected in these samples. The proportions of bacteria previously shown to be associated with CRC were differentially represented in tumoral crypts from right and left colonic samples. For instance, *Fusobacterium* and Bacteroides fragilis were abundant in tumors from the right colon, whereas Parvimonas micra was associated with tumors from the left colon. In healthy samples, *Faecalibacterium* was more abundant in right than in left colonic crypts. Taken together, our results demonstrate the existence of a human CSCM and point to a specific localization of bacteria previously associated with CRC. The presence of an abnormal microbiota in colonic crypts is hypothesized to be linked to CRC oncogenesis, but further studies are needed to explore this association.

## RESULTS

### Eligible subjects and samples characteristics.

A total of 67 subjects, 9 healthy volunteers (1 man and 8 women) and 58 CRC patients (37 men and 21 women), were included in the study. Patient characteristics are shown in Table 1. Briefly, the range of ages was from 23 to 92 years, with a median of 70 years, and the body mass index was from 16 to 44.7, with a median of 25.8. Samples were divided into 29 right colon, 34 left colon, and 4 rectal cancers. Rectal cancer samples were excluded because of low sample size and thus low statistical power. Patients under chemotherapy, radiotherapy, or antibiotic treatment were excluded from the present study. The study was approved by the Comité de Protection des Personnes, by the Comité Consultatif pour le Traitement de l’Information en Matière de Recherche dans le Domaine de la Santé, and by the Commission Nationale de l’Informatique et des Libertés (project 2012-37). Patients provided written informed consent for the collection of samples and subsequent analysis. The right and left colon surgical biopsy specimens were obtained without prior cleansing, while for rectal samples and those collected during colonoscopies, patients underwent a protocol that included colon cleansing, anesthesia, and colonoscopy procedures, and none of the patients had received antibiotics within 4 weeks prior to colonoscopy or surgery.

**TABLE 1:** Patient characteristics

Patient	Gender^*a*^	Age (yr)	Wt (kg)	Ht (cm)	BMI^*b*^	Organ	Tumor site	Stage^*c*^	T/N/M^*c*^
1	F	77	113	159	44.70	Colon	Right	2	3/0/0
2	F	54	75	164	27.89	Colon	Right	3	3/2a/0
3	F	89	82	170	28.37	Colon	Right	2	3/0/0
4	M	84	75	169	26.26	Colon	Left	2	3/0/0
5	F	75	49	164	18.22	Colon	Right	3	4b/0/0
6	M	88	65	176	20.98	Rectum	Rectum	1	2/0/0
7	F	52	95	176	30.67	Colon	Left	0	3/1a/0
8	M	90	78	171	26.67	Colon	Left	2	3/0/0
9	M	87	120	171	41.04	Colon	Left	2	4/0/0
10	M	23	76	177	24.26	Colon	Left	3	3/1a/0
11	M	67	100	180	30.86	Colon	Right	4	3/1c/1
12	F	85	60	153	25.63	Rectum	Rectum	1	2/0/0
13	M	78	85	173	28.40	Colon	Left	4	3/1c/1
14	M	85	63	168	22.32	Rectum	Rectum	1	1/0/0
15	M	62	92	171	31.46	Colon	Left	2	3/0/0
16	F	75	63	163	23.71	Colon	Right	1	2/0/0
17	M	57	78	176	25.18	Colon	Left	0	3/0/0
18	F	28	58	173	19.38	Colon	Right	3	4b/0/0
19	M	66	86	170	29.76	Colon	Right	2	3/0/0
20	F	57	62	155	25.81	Colon	Right	2	3/0/0
21	M	61	62	168	21.97	Colon	Left	0	3/0/0
22	F	92	85	160	33.20	Colon	Left	3	3/1b/0
23	F	77	62	158	24.84	Colon	Left	1	2/0/0
24	M	60	79	175	25.80	Colon	Left	4	4b/2b/1
25	M	84	68	175	22.00	Colon	Right	2	3/0/0
26	M	84	69	152	29.86	Colon	Left	2	4/0/0
27	M	64	65	168	23.03	Colon	Right	1	1/0/0
28	M	45	112	183	33.44	Colon	Left	0	0/0/0
29	M	67	76	175	24.82	Colon	Right	4	4/0/1
30	M	78	64	169	22.41	Colon	Left	2	3/0/0
31	F	75	61	160	23.83	Colon	Right	3	3/1b/0
32	M	66	70	172	23.66	Rectum	Rectum	3	4/0/0
33	M	89	95	177	30.32	Colon	Left	0	3/0/0
34	M	72	81	175	26.45	Colon	Right	4	4/2b/1
35	F	60	70	149	31.53	Colon	Left	3	3/2a/0
36	M	70	71	167	25.46	Colon	Right	2	3/0/0
37	M	51	70	174	23.12	Colon	Left	3	3/1a/0
38	M	52	61	160	23.83	Colon	Left	3	3/1/0
39	M	71	86	180	26.54	Colon	Left	2	3/0/0
40	M	76	94	178	29.67	Colon	Right	3	3/2b/0
41	M	74	86	168	30.47	Colon	Left	3	3/1a/0
42	F	52	70	165	25.71	Colon	Right	1	1/0/0
43	M	88	67	171	22.91	Colon	Right	3	3/1a/0
44	M	70	100	173	33.41	Colon	Right	0	1/0/0
45	F	72	71	163	26.72	Colon	Right	0	1/0/0
46	F	86	66.5	167	23.84	Colon	Right	4	4/1b/1
47	M	58	56	167	20.08	Colon	Right	3	3/1b/0
48	M	85	77	175	25.14	Colon	Left	2	3/0/0
49	M	71	73	175	23.84	Colon	Right	2	3/0/0
50	M	71	62	178	19.57	Colon	Left	4	4/1a/1
51	F	89	71	170	24.57	Colon	Right	4	3/0/0
52	F	25	42	162	16.00	Colon	Left	3	4b/2a/0
53	F	76	95	157	38.54	Colon	Right	2	3/0/0
54	M	61	70	175	22.86	Colon	Left	4	4/1b/1
55	M	75	60	168	21.30	Colon	Left	2	3/0/0
56	M	60	83	174	27.41	Colon	Left	0	0/0/0
57	F	47	76	170	26.30	Colon	Right	0	0/0/0
58	F	68	57	153	24.30	Colon	Right	0	0/0/0

S1	F	52	88	170	26.30	Colon	Left	0	
S2	F	81	74	164	27.51	Colon	Left	0	
S3	F	29	68	162	25.91	Colon	Left	0	
S4	M	55	130	176	41.97	Colon	Right	0	
S5	F	29	68	162	25.91	Colon	Left	0	
S6	F	54	63	163	23.71	Colon	Left	0	
S7	F	34	68	158	27.24	Colon	Right	0	
S8	F	38	78	179	24.34	Colon	Left	0	
S9	F	56	74	168	26.22	Colon	Left	0	

a F, female; M, male.

b BMI, body mass index (body mass divided by the square of the body height) expressed in kilograms per square meter.

c The earliest stage colorectal cancers are called stage 0, and then they range from stages 1 to 4. The lower the number, the less the cancer has spread, and within a stage, an earlier letter means a lower stage. T/N/M, classifications of tumors, where T is the size of the tumor, N indicates whether lymph nodes are involved, and M indicates distant metastasis.

### Sequencing results.

All samples were sequenced on four Illumina MiSeq runs. As mentioned in Materials and Methods, different controls were included in each run to detect possible contamination in water or extraction buffer used during microdissection, DNA extraction, and the 16S rRNA gene PCR amplification. For each control, a library was prepared and sequenced. These control samples after preprocessing steps yielded between 1 and 366 sequences per sample, with a mean of 98 and a median of 67.5 sequences. The numbers of sequences obtained for the microdissected samples as well as the relative abundance for each taxon are reported in Table S1A in the supplemental material. Alpha diversity was estimated using the Chao1 index, which indicates the richness of bacterial communities based on the abundance of rare species belonging to each group of samples. The rarefaction plot indicates that the bacterial compositions of the samples from nontumoral colonic tissue from CRC patients has a higher richness than those from tumoral colonic tissues (Fig. S1A). As mentioned in Materials and Methods, two databases were used to perform taxonomic assignment of each operational taxonomic unit (OTU): Greengenes (35) and HITdb (36). The comparative analyses between cancerous and noncancerous samples were performed using HITdb, as this database permits taxonomic analysis to the species level. The corresponding OTU assignments from phylum to species, obtained with HITdb, are reported in Table S1B.

10.1128/mBio.01315-19.1 FIG S1 (A) Alpha diversities estimated using the Chao1 index. The rarefaction plot shows Chao1 measures of richness of bacterial communities between tumoral (T) and nontumoral (NT) tissues. Principal-coordinate analysis (PCoA) plot showing microbiota community clusters determined by unweighted UniFrac analysis. (B) Crypt and mucosa-associated samples. (C) CAM samples. (D) MAM samples. White dots represent nontumoral samples, whereas red dots represent tumoral samples. Download FIG S1, JPG file, 0.4 MB. Copyright © 2019 Saffarian et al. 2019 Saffarian et al. This content is distributed under the terms of the Creative Commons Attribution 4.0 International license.

10.1128/mBio.01315-19.7 TABLE S1 Data set of the patient samples, including the identification of the sample and the number of reads in one tab (mapping table) and the relative abundance of each taxon in the other tab (relative abundance) of the Excel file (S1A). OTU assignments from the phylum to the species level according to the HITdb database (S1B). Download Table S1, XLSX file, 2.8 MB. Copyright © 2019 Saffarian et al. 2019 Saffarian et al. This content is distributed under the terms of the Creative Commons Attribution 4.0 International license.

### Core microbiotas in normal colonic tissues.

**FIG 1:**
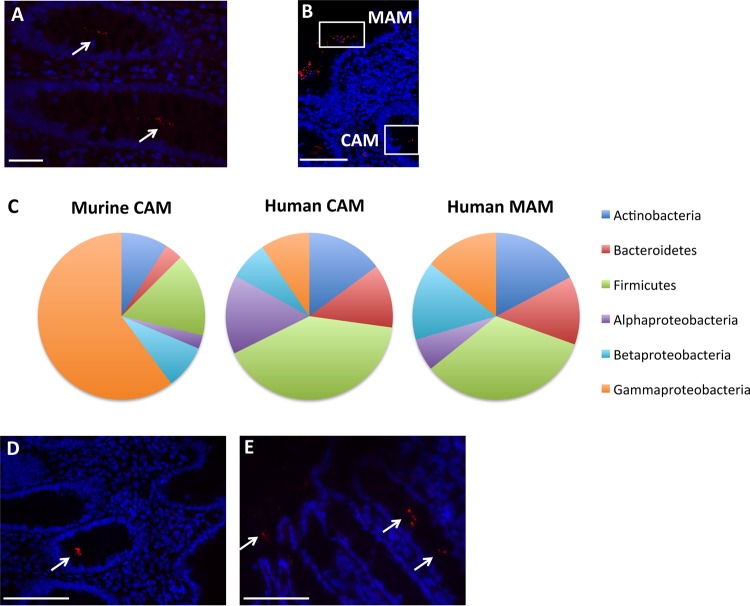
Microbiotas of control subjects. (A and B) Representative images from FISH analyses with the pan-bacterial probe Eub338 (red) of crypt-associated microbiota (CAM) (A) and mucosa-associated microbiota (MAM) (B) observed in normal colonic biopsy specimens. Panel B includes a representation of the CAM and MAM regions. (C) Average relative abundances at the phylum level in murine CAM and in human CAM and MAM. (D and E) Representative pictures from FISH analyses with the Acinetobacter-specific probe (red) of CAM (D) and MAM (E) in normal colonic biopsy specimens. White arrows indicate the presence of bacteria. Nuclei are counterstained with DAPI (blue). Scale bars: 20 μm (A) and 50 μm (B, D, and E).

The study aimed to evaluate whether bacteria could be detected in human colonic crypts, as has previously been described for mice (34). Using normal colonic biopsy specimens, bacteria were visualized inside the intestinal crypts by fluorescence *in situ* hybridization (FISH) technology utilizing a pan-bacterial probe (Fig. 1A), as well as in the mucosa-associated region of the two right and seven left colon biopsy specimens (Fig. 1B). These bacteria were then identified by 16S rRNA sequencing performed on LCM samples of the CAM and MAM regions, as illustrated in Fig. 1B. *Firmicutes* and *Proteobacteria* were the most abundant phyla present in both sections, with average relative abundances of 36.96% and 35.1% in CAM and 33.29% and 35.43% in MAM, respectively. *Actinobacteria* and *Bacteroidetes* were also detected at lower levels, with average abundances of 15.22% and 12% in CAM and 17.04% and 13.03% in MAM, respectively. Among the *Proteobacteria*, which were in even lower abundances than in proximal murine colonic crypts, Acinetobacter (4.27% and 6% in CAM and MAM, respectively), *Delftia* (2.13% and 3.66% in CAM and MAM, respectively), and *Stenotrophomonas* (1.55% and 3.7% in CAM and MAM, respectively) were also present in human colonic tissue (Fig. 1C; Table S1A). In addition, *Paracoccus* and *Sphingomonas*, belonging to the *Alphaproteobacteria* class, and *Ralstonia* and *Acidovorax*, belonging to the *Betaproteobacteria* class, were present in human control colonic crypts (Table S1A). This demonstrated that, similarly to murine proximal colon crypts, human colonic crypts harbor Gram-negative, aerobic, nonfermenting, environmental, highly biodegradative *Proteobacteria*. We further validated the presence of Acinetobacter in both CAM and MAM by FISH using a probe specific to this genus (Fig. 1D and E). The common core microbiota was defined as being shared in more than 50% of subjects in each group (37, 38). Using this criterion, 28 OTUs were common to the control CAM and MAM, whereas 22 OTUs were present only in CAM and 3 OTUs were specific to mucosa-associated samples (Table S2A). In addition to Acinetobacter junii, *Delftia*, and Stenotrophomonas maltophilia, OTUs assigned to *Corynebacterium*, Kocuria palustris, *Blautia*, and *Roseburia* were shared between normal CAM and MAM. The OTU assigned to Faecalibacterium prausnitzii was found only in the core of MAM.

10.1128/mBio.01315-19.8 TABLE S2 Core OTU of biopsy specimens from control patients (S2A) and from CRC patients (S2B). The different categories are listed in the tab “Category” of the Excel sheet, where “P” means the presence of the OTU and “A” indicates its absence. Download Table S2, XLSX file, 0.1 MB. Copyright © 2019 Saffarian et al. 2019 Saffarian et al. This content is distributed under the terms of the Creative Commons Attribution 4.0 International license.

### Phylum- and order-level compositions of the microbiotas associated with CRC colonic tissues.

Representative crypt hematoxylin-eosin images of normal (Fig. S2A) or cancerous (Fig. S2D) tissues are presented in the supplemental material. Aberrant crypts are clearly visualized in the tumoral tissue (Fig. S2D). The presence of bacteria inside the crypts of cancerous and noncancerous tissues was confirmed by FISH using a pan-bacterial probe targeting 16S rRNA (Fig. S2B and E). This result indicates that bacteria can be detected inside the crypts of nontumor and tumor tissues from CRC patients. These findings are in line with the above-mentioned presence of bacteria in normal biopsy specimen tissue (Fig. 1A), as reported in a recent publication analyzing the crypts from a patient with a right-side colon cancer (39). Representative images of LCM sections of crypts from homologous normal and tumor sites are shown in Fig. S2C and F, respectively. The region immediately adjacent to the tissue was also microdissected to extract the DNA of MAM, as was performed for the control biopsy specimens.

10.1128/mBio.01315-19.2 FIG S2 Comparison of homologous normal and cancerous tissues. (A) Hematoxylin-eosin staining of homologous normal colonic tissue. (B) Representative picture of FISH analyses with the pan-bacterial probe Eub338 (red) of normal colonic tissue. Nuclei stained with DAPI (blue). (C) Representative image of a laser-captured normal colonic tissue. (D) Hematoxylin-eosin staining of tumoral colonic tissue from the same CRC patient. (E) Representative picture of FISH analyses with the pan-bacterial probe Eub338 (red) of tumoral colonic tissue. Nuclei are counterstained with DAPI (blue). (F) Representative picture of a laser-captured tumoral colonic tissue. Scale bars: 400 μm (A, C, D, and F) and 50 μm (B and E). Download FIG S2, JPG file, 0.5 MB. Copyright © 2019 Saffarian et al. 2019 Saffarian et al. This content is distributed under the terms of the Creative Commons Attribution 4.0 International license.

As reported by others (4, 40, 41), the human colonic microbial composition showed high interindividual variability. For example, in the present study, *Firmicutes* represented between 0.4 and 74.6% (mean, 24.85%) and 0.4 and 88.3% (mean 27.31%) of bacteria in crypt samples and mucosa-associated samples, respectively (Tables S1A and B). The interindividual variability in right and left colon specimens from CAM and MAM are shown in Fig. S3. The phylum *Bacteroidetes* accounted for between 0 and 52.1% (mean, 16.16%) of bacteria in crypt samples and for between 0 and 47.5% (mean 25.04%) of bacteria in mucosa-associated samples. In addition, unweighted UniFrac principal-component analysis (PCoA) analysis showed a cluster overlap of the microbiotas from tumoral and adjacent nontumoral tissues (Fig. S1B). This overlap was further observed at the crypt level (Fig. S1C) and also at the mucosa-associated level (Fig. S1D), indicating that there is no overall significant difference in microbiota compositions between tumoral and healthy homologous colon samples, as already reported in other studies (7, 24). However, despite the observed patient individual variability, differences between paired tumor and normal samples were observed (Table S1B), indicating that, in each individual, microbiota composition was modified in the colonic tumor environment compared to that of the normal adjacent tissue.

10.1128/mBio.01315-19.3 FIG S3 Individual relative abundances at the phylum level in CAM and MAM. (A) Right CAM; (B) left CAM; (C) right MAM; (D) left MAM. The numbers indicate the identifications of the patient. N, nontumoral; T, tumoral. Download FIG S3, JPG file, 0.8 MB. Copyright © 2019 Saffarian et al. 2019 Saffarian et al. This content is distributed under the terms of the Creative Commons Attribution 4.0 International license.

For example, *Fusobacterium* was more abundant in tumors than in paired nontumor samples. At the order level, *Fusobacteriales* accounted for 0.1 to 4.5% of the organisms in nontumoral samples, with an average of 1.06%, and for 0.1 to 86.6% in tumor samples, with an average of 6.27%. For the patient with a *Fusobacterium* abundance of 86.6% in the tumor crypt sample, an abundance of 26.3% was found in the mucosa-associated sample, whereas in the patient’s nontumor samples, the abundances were, respectively, 0.3 and 1.1% in the crypt and in the mucosa-associated samples.

### Species-level composition of the microbiota associated with CRC colonic tissues.

**FIG 2:**
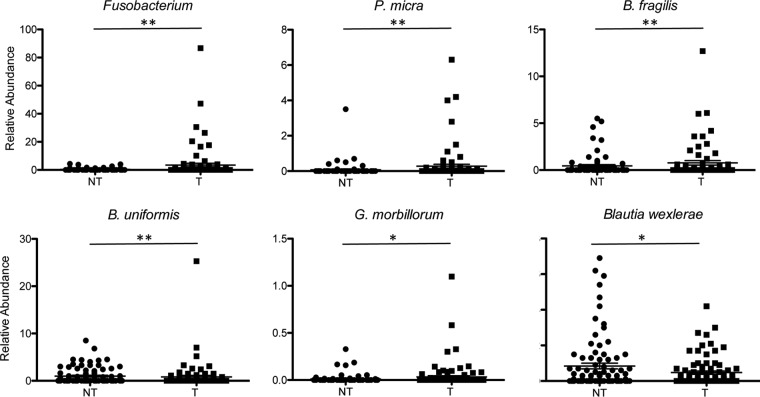
Relative abundances of selected bacterial species in tumoral (T) and nontumoral (NT) samples. The percentages represent the sums of the relative abundances found in CAM and MAM. Data are displayed as means ± standard errors of the means (SEM) and were analyzed by the fitZIG test. *, *P < *0.05; **, *P < *0.01.

Among the *Fusobacteriaceae* family, Fusobacterium periodonticum and Leptotrichia buccalis were the most abundant species observed in cancerous samples and often found together in the same samples. Fusobacterium nucleatum was found in the mucosa-associated sample of two CRC patients and in association with *F. periodonticum* and B. fragilis (Table S1A). A nonparametric Kruskal-Wallis test and a zero-inflated Gaussian (ZIG) mixture model (42–44) identified several taxa with significantly different relative abundances between tumor and adjacent nontumor samples, including *Fusobacterium*, Bacteroides fragilis, and Gemella morbillorum (Table S3). The ZIG mixture model was used in subsequent analyses. Parvimonas micra (*P* = 0.00822), *F. periodonticum* (*P* = 0.01626), Bacteroides uniformis (*P* = 0.00547), and *G. morbillorum* (*P* = 0.01599) were more abundant in tumor samples than in adjacent noncancerous tissues. Coassociation of these bacteria was not necessarily observed in the same tumor tissue. For example, for the three patients with the highest abundance of *P. micra*, *F. periodonticum* and B. fragilis were also associated in patient 4, whereas B. fragilis was present in patient 48 and *F. periodonticum* was present in patient 8 (Table S1A). Even if B. fragilis could be detected in both tumor and nontumor samples, the 38 OTUs assigned to B. fragilis indicate a significant increase of its relative abundance in tumor samples compared to in nontumor samples (*P* values are from 0.00191 to 0.04981). Some OTUs assigned to B. fragilis were present in tumoral CAM and absent in tumoral MAM and vice versa for other OTUs (Table S3B). *S. gallolyticus* is present only in low numbers of samples (15 tumor and 5 nontumor samples) and at very low abundances (from 0.001 to 0.11%). However, there is a significant increase in the number of OTUs assigned to *S. gallolyticus* in tumor samples versus adjacent nontumor samples (*P* = 0.01296). In contrast, Blautia wexlerae is less represented in cancer samples than in noncancer samples (*P* = 0.04424). The relative abundances of selected species in nontumoral and tumoral samples are represented in Fig. 2.

10.1128/mBio.01315-19.9 TABLE S3 (S3A) Group significance analysis using the Kruskal-Wallis test implemented in QIIME. Only *P* values of <0.05 are shown. T versus NT, tumoral samples versus nontumoral samples; CAM T versus NT, crypt-associated microbiota from tumoral samples versus crypt-associated microbiota from nontumoral samples; MAM T versus NT, mucosa-associated microbiota from tumoral samples versus mucosa-associated microbiota from nontumoral samples. (S3B) Differential analysis using fitZIG at the species level. Only *P* values of <0.05 are shown. NT versus T, nontumoral samples versus tumoral samples; CAM NT versus T, crypt-associated microbiota from nontumoral samples versus crypt-associated microbiota from tumoral samples; CAM NT (right to left), crypt-associated microbiota from nontumoral samples, right colon versus left colon; CAM T (right to left), crypt-associated microbiota from tumoral samples, right colon versus left colon; CAM right NT versus T, crypt-associated microbiota from right colon, nontumoral versus tumoral samples; CAM left NT versus T, crypt-associated microbiota from left colon, nontumoral versus tumoral samples; MAM T versus NT, mucosa-associated microbiota from tumoral samples versus mucosa-associated microbiota from nontumoral samples; MAM NT (right to left), mucosa-associated microbiota from nontumoral samples, right colon versus left colon; MAM T (right to left), mucosa-associated microbiota from tumoral samples, right colon versus left colon; MAM right T versus NT, mucosa-associated microbiota from right colon, tumoral versus nontumoral samples; MAM left T versus NT, mucosa-associated microbiota from left colon, tumoral versus nontumoral samples; NT CAM versus MAM, crypt-associated microbiota versus mucosa-associated microbiota in nontumoral samples; T CAM versus MAM, crypt-associated microbiota versus mucosa-associated microbiota in tumoral samples. Download Table S3, XLSX file, 2.4 MB. Copyright © 2019 Saffarian et al. 2019 Saffarian et al. This content is distributed under the terms of the Creative Commons Attribution 4.0 International license.

### qPCR and FISH analyses.

**FIG 3:**
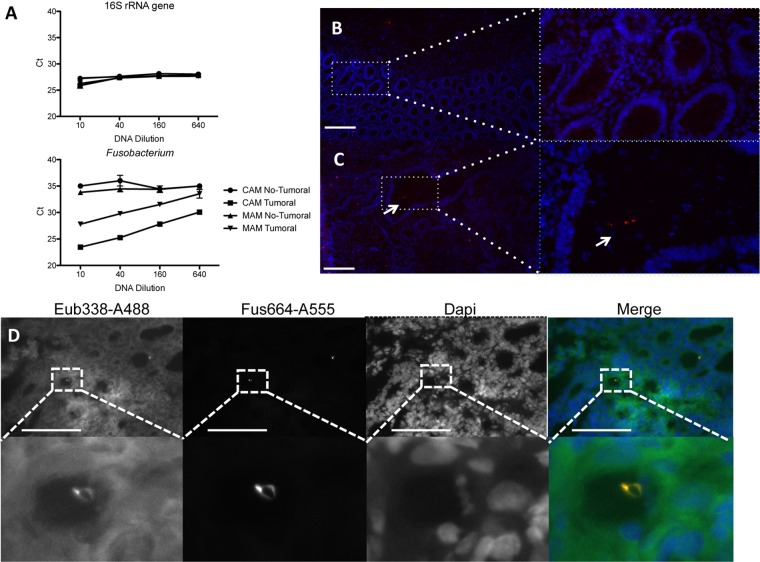
Validation of the presence of *Fusobacterium* in colonic tissues. The results of qPCR amplification of microdissected DNA are shown. (A) Amplification of microdissected samples using 16S rRNA genes or *Fusobacterium* primers. (B and C) Images are representative of FISH analyses with a *Fusobacterium*-specific probe linked to Alexa 555 of the homologous normal tissue (B) or the paired tumoral colonic tissue (C) of the same patient. (D) Representative images of FISH analyses with the pan-bacterial probe Eub338 (green) and the *Fusobacterium*-specific probe (red). Nuclei are stained in blue with DAPI. Scale bars: 100 μm (B and D) and 20 μm (C).

16S rRNA gene sequencing results were validated by quantitative PCR (qPCR) using primers specific for the 16S rRNA gene of *Fusobacterium* genus, B. fragilis, and *P. micra* and by FISH using fluorescent probes specific for the 16S rRNA of the *Fusobacterium* genus and B. fragilis. In addition, primers targeting a conserved region on the 16S rRNA gene were also used for the PCR to quantify the bacterial DNA present in each sample. Water served as the template control in order to determine the threshold of detection, while dilution series of genomic DNA from E. coli, Fusobacterium nucleatum subsp. *animalis*, B. fragilis, and *P. micra*, in water or in extracted human tissue (spike-in controls), were used to test the specificities of the primer pairs. The threshold cycle (*C_t_*) values of the water control were 27.33 ± 0.10 with the 16S pan-bacterial primers, 35.25 ± 0.48 with the *Fusobacterium* primers, 32.15 ± 0.27 with the B. fragilis primers, and 36.51 ± 1.32 with the *P. micra* primers. The results shown in Fig. S4A and B confirm that the primers used were specific to their corresponding genomic DNA and that the presence of eukaryotic DNA did not inhibit amplification. *P. micra* DNA present in the microdissected samples was found in insufficient quantities for PCR amplification, which has a lower sensitivity than 16 rRNA gene sequencing. However, using the *Fusobacterium* primers, we were able to confirm the 16S rRNA gene sequencing results. The abundance of *Fusobacterium* DNA was higher in samples from crypt-associated regions and mucosa-associated regions from tumor tissue than in the samples from noncancer tissue from the same patient (Fig. 3A). Using a fluorescent probe targeting the 16S rRNA of *Fusobacterium*, bacteria could be observed in the tumor crypt (Fig. 3C) but not in homologous normal tissue (Fig. 3B). The presence of *Fusobacterium* in tissue from another cancer patient was also visualized by FISH using a combination of the *Fusobacterium*-specific probe and the probe targeting a constant region of the bacterial 16S rRNA (Fig. 3D). To obtain a higher-resolution taxonomic characterization of *Fusobacterium*, we amplified and sequenced the *Fusobacterium rpoB* gene in crypt samples. A BLASTN analysis of the PCR product sequences identified F. nucleatum subsp. *polymorphum*.

10.1128/mBio.01315-19.4 FIG S4 Test of the specificity of the primers used for qPCR. The sequences of the primers are listed (data not shown). Serial dilutions of genomic bacterial DNA were used as a template with the following primer couples: *Fusobacterium*, B. fragilis, and *P. micra*. Bacterial genomic DNAs were diluted in water (A) or with an extract of human colonic tissue (B). Download FIG S4, JPG file, 0.4 MB. Copyright © 2019 Saffarian et al. 2019 Saffarian et al. This content is distributed under the terms of the Creative Commons Attribution 4.0 International license.

**FIG 4:**
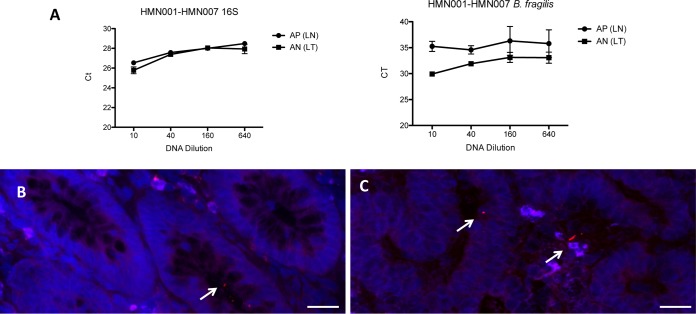
Validation of the presence of Bacteroides fragilis in colonic tissues. qPCR amplification of microdissected DNA is shown. (A) Amplification of microdissected samples using 16S rRNA genes or B. fragilis primers. AP and AN represent the LCM identification of samples from the nontumoral (LN) and tumoral (LT) MAM regions. (B and C) Images are representative of FISH analyses with a B. fragilis-specific probe linked to Alexa 555 of a noncancerous tissue (B) or the paired tumoral colonic tissue (C) of the same patient. Nuclei are counterstained in blue with DAPI. Scale bars: 20 μm.

An example of PCR amplification of microdissected samples shows also that the abundance of B. fragilis DNA is higher in sample AN from a tumor mucosa-associated region than in the paired normal mucosa-associated region (sample AP), confirming the sequencing results (Fig. 4A). The presence *of* B. fragilis inside colonic crypts could also be validated by FISH using a fluorescent probe targeting the 16S rRNA of B. fragilis in both nontumor (Fig. 4B) and tumor (Fig. 4C) crypts. The presence of the *bft* gene, encoding the enterotoxin of B. fragilis, was not detected by PCR in the samples positive for the presence of B. fragilis 16S rRNA genes.

### Core bacteria in CRC patients.

As the microbial communities of a tumor and paired normal tissue from a given patient significantly differed from each other, core OTUs were defined as present in more than 50% of individuals, as was done for control samples. As shown in Fig. S5 and in Table S2B, 26 OTUs were shared by the four categories of cancerous patients. OTUs assigned to *Proteobacteria*, such as *Paracoccus*, *Acidovorax*, Acinetobacter junii, *Delftia*, and S. maltophilia, belonged to this common core, similarly to control samples. Two other OTUs assigned to *A. jun*ii were specifically found in tumor CAM. One OTU (denovo 354529) of *F. prausnitzii* was found in the core of control MAM samples and was shared between the four categories of cancer samples, whereas another one (denovo 180406) was found in nontumoral CAM and MAM. Only three OTUs were specific to tumoral CAM and MAM samples and were assigned to Prevotella copri, Staphylococcus hominis, and Comamonas kerstersii.

10.1128/mBio.01315-19.5 FIG S5 Venn diagram of the number of OTUs present in the core OTU of each group. CAM NT, crypt-associated microbiota from nontumoral samples; CAM T, crypt-associated microbiota from tumoral samples; MAM NT, mucosa-associated microbiota from nontumoral samples; MAM T, mucosa-associated microbiota from tumoral samples. Download FIG S5, JPG file, 0.2 MB. Copyright © 2019 Saffarian et al. 2019 Saffarian et al. This content is distributed under the terms of the Creative Commons Attribution 4.0 International license.

### Bacterial community in cancerous tissue and adjacent noncancerous normal crypts from right and left colon specimens.

**FIG 5:**
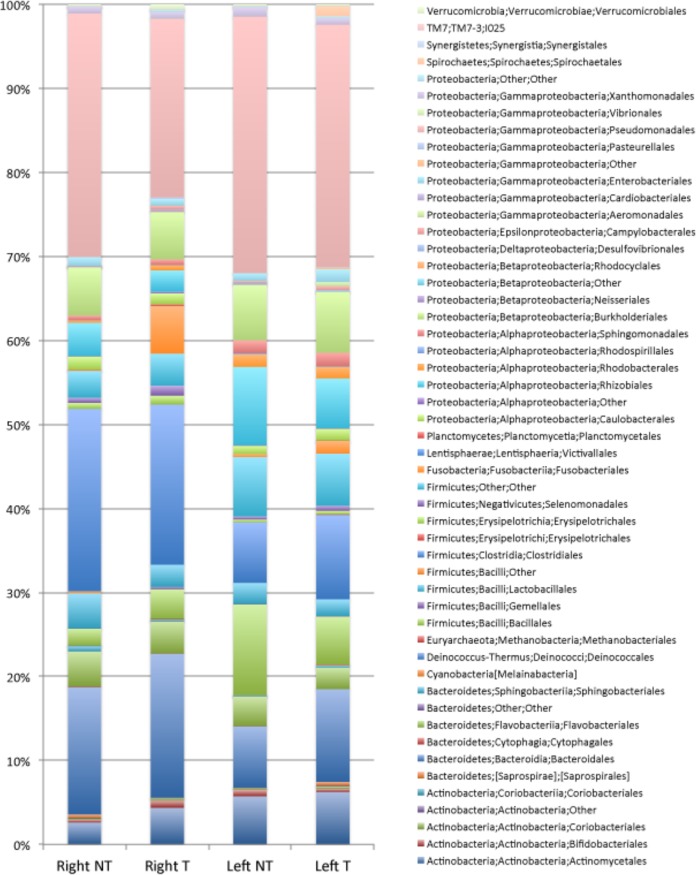
Average relative abundances at the order level in CAM. Percentages are from the normal right colon (Right NT) or normal left colon (Left NT) and from the tumoral right colon (Right T) and tumoral left colon (Left T).

**FIG 6:**
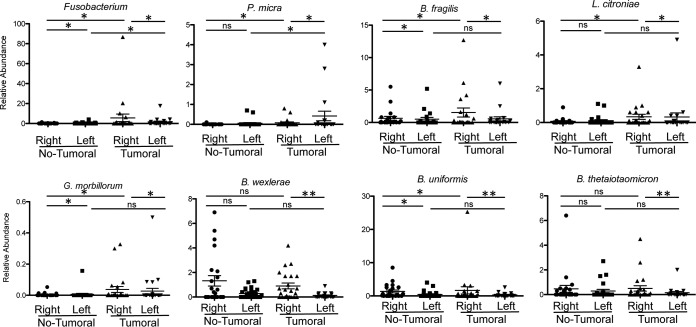
Relative abundances of selected bacterial species in crypt samples from the right colon and left colon from nontumoral and tumoral samples. Data are means ± SEM and were analyzed by the fitZIG test. *, *P < *0.05; **, *P < *0.01; ns, differences were not significant.

Globally, at the global crypt level, our findings show greater relative abundances of *P. micra* (OTU 121066, *P* = 0.00103) and *F. periodonticum* (OTU 178918, *P* = 0.00622) when tumoral samples are compared to the adjacent nontumoral samples (Table S3B). Similar findings were observed for *G. morbillorum* (OTU 158176, *P* = 0.00823), *Lachnoclostridium citroniae* (OTU 172457, *P* = 0.04695), and Peptostreptococcus stomatis (OTU 149267, *P* = 0.03663). To evaluate if the observed differences exist in both the right and the left colon, we compared the relative abundances of these OTUs between the two separate sites. As shown in Fig. 5, the relative abundances of these taxa differ between the right and left colons even in noncancerous crypts. For example, *Bacteroidiales* and *Clostridiales* were more abundant in nontumoral crypt samples from right colons than in samples from the left colons, whereas the opposite was observed for *Bacillales*. More precisely, OTU denovo 162624, assigned to Eubacterium rectale (*P* = 0.04264), and OTU denovo 37653, assigned to *Ruminococcaceae* (*P* = 0.01713), were more abundant in right noncancerous colon specimens than in left noncancerous colon specimens, whereas *Paracoccus* (OTU 167461, *P* = 0.00927) was more abundant in left noncancerous colon than in right noncancerous colon (Table S3B). The relative abundances of *F. periodonticum*, *P. micra*, *L. citroniae*, and B. fragilis in the tumoral crypts from the right and left colon specimens showed marked differences. While *Fusobacterium*, B. fragilis, and *L. citroniae* were more abundant in right tumoral crypts than in left tumoral crypts, *P. micra* was more abundant in left tumoral crypts (Fig. 6 and Table S3B). Moreover, *B. uniformis* (OTU 164580, *P* = 0.02282), *B. wexlerae* (OTU 59724, *P* = 0.00362), and *E. rectale* (OTU 162624, *P* = 0.00208) were more abundant in crypts in cancerous right colons than from cancerous left colons. *F. periodonticum* is more abundant in crypts from cancerous samples from right colons than in nontumoral adjacent samples (*P* = 0.010007), as with *P. micra* (*P* = 0.001939) and *G. morbillorum* (*P* = 0.022105). However, this was not the case for *G. morbillorum* in left colonic samples (Fig. 6 and Table S3B). Lastly, OTU 64963, assigned to Acinetobacter lwoffii, was more abundant in crypts from nontumoral samples from the right colons than from the samples’ paired tumoral counterparts.

### Bacterial communities in cancerous mucosa-associated regions and adjacent noncancerous mucosa-associated regions from right and left colons.

**FIG 7:**
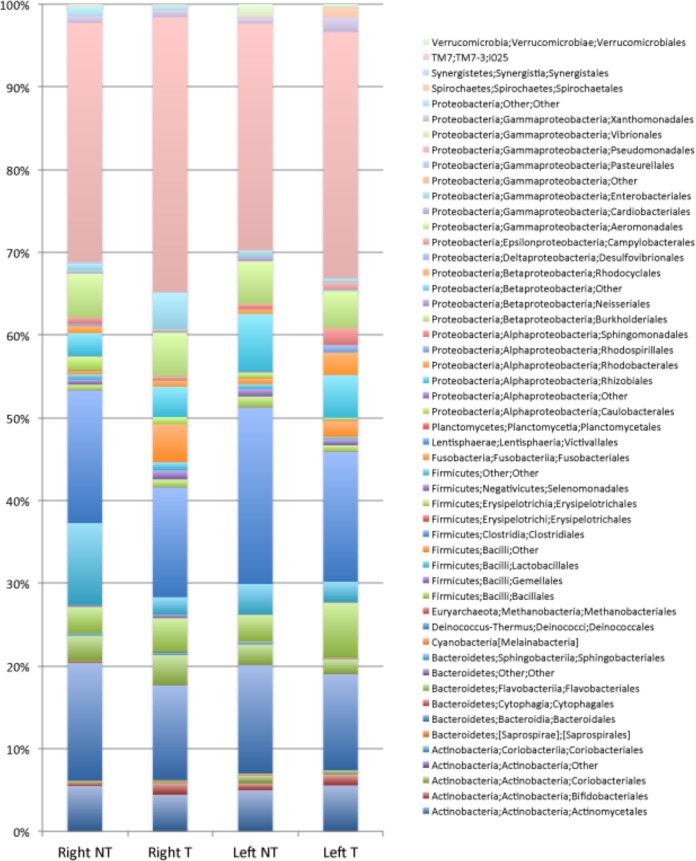
Average relative abundances at the order level in MAM. Percentages are from the normal right colon (Right NT) or normal left colon (Left NT) and from the tumoral right colon (Right T) and tumoral left colon (Left T).

**FIG 8:**
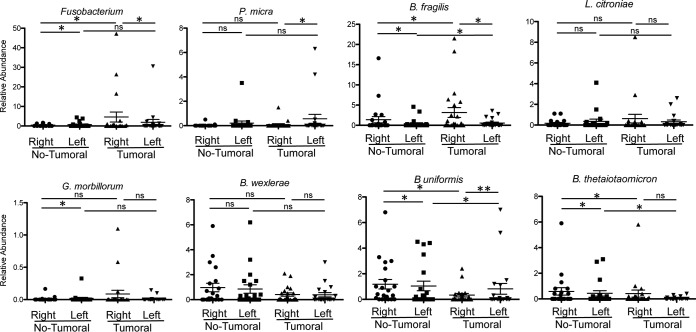
Relative abundances of selected bacterial species in mucosa samples from the right colon and left colon from nontumoral and tumoral samples. Data are means ± SEM and were analyzed by the fitZIG test. *, *P < *0.05; **, *P < *0.01; ns, differences were not significant.

To assess if the observed differences in the crypt also occurred in the mucosa-associated samples, we compared the average relative abundances of mucosa-associated bacteria in right and left colonic samples, as presented in Fig. 7. A slight increase in the relative abundance of *Fusobacteria* (*F. periodonticum* and *Leptotrichia*) was observed in tumor samples compared to abundances in the paired, noncancerous samples; however, the differences were not significant (*P* values, 0.366263 and 0.154968, respectively). On the other hand, Bacteroides thetaiotaomicron (OTU 185118) and *B. uniformis* were significantly more abundant in mucosa-associated noncancerous samples than in mucosa-associated cancerous samples (*P* values, 0.016581 and 0.013195, respectively) (Fig. 8). Furthermore, Streptococcus vestibularis (*P* = 0.03064), Streptococcus oralis (*P* = 0.023305), Moraxella osloensis (*P* = 0.016988), and Kocuria palustris (*P* = 0.010586) were more abundant in noncancerous samples from the right colon than from the left colon. For example, *K. palustris* accounted for 0.1 to 13.2% of mucosa-associated bacteria in the right colon (mean, 1.72%; 12 positive samples out of 21) and for 0.1 to 0.7% of mucosa-associated bacteria in left colon samples (mean, 0.12%; 8 positive samples out of 20). At the species level, in the tumoral samples, no OTU had significantly different relative abundances in MAM from right and left colon samples. As for the crypt samples, there are statistically significant differences in the relative abundances of *F. periodonticum*, *P. micra*, and B. fragilis in the mucosa-associated microbiotas from right and left cancerous colonic samples, whereas this is not the case for *L. citroniae*. Moreover, in contrast to the results observed for the crypt samples, there are no statistically significant differences in *P*. *micra* and *G. morbillorum* abundances between mucosa samples from cancerous samples from the right colon and mucosa samples from nontumoral samples (Fig. 8 and Table S3B).

## DISCUSSION

Most studies addressing the relationship between the microbiota and CRC have been based on an analysis of the bacterial compositions of either fecal samples or, to a lesser extent, tumoral tissues and their paired adjacent normal tissues. In the present study, we performed an in-depth analysis of the bacterial compositions in colonic crypts and mucosa-associated compartments in both CRC patients and individuals with normal colonoscopy results. First, we confirmed that colonic crypts in healthy volunteers are colonized by bacteria. While this result supports earlier published findings (45), another group reported that no bacteria were found in colonic crypts of healthy individuals (46). The combination of LCM and 16S rRNA gene sequencing applied in this study allowed for the identification of bacterial species colonizing the crypts as well as those associated with the mucosa. Interestingly, the three genera (Acinetobacter, *Delftia*, and *Stenotrophomonas*) which we previously described as the crypt-specific core microbiota in murine proximal colon crypts (34) were also retrieved in human normal colonic crypts, and OTUs associated with these genera belong to the common core microbiota. These bacteria were also found in crypts and mucosa-associated regions from tumor sites and their paired adjacent normal tissue, and we did not observe a significant decrease in the abundance of Acinetobacter in tumoral samples compared to that in adjacent normal tissue, unlike results reported by other studies (29, 47). Instead, we identified additional Gram-negative, aerobic, nonfermentative, environmental *Proteobacteria* (such as *Ralstonia* and *Acidovorax*) that inhabit human colonic crypts. It is interesting to note that *Proteobacteria*, such as *Comamonadaceae*, *Moraxellaceae*, *Pseudomonadaceae*, and *Xanthomonadaceae*, represent 31.9% and 35.2% of CAM and MAM, respectively, whereas the abundances of these phyla represent only 1% of the fecal microbiota (48). Taken together, the present study was able to extend the concept of a human crypt-specific core microbiota (CSCM), which has so far been established only in rodents. Like its murine counterpart, human CSCM is marked by the strong representation of strictly aerobic and facultative anaerobic taxa that are likely to be part of a coevolutionary symbiosis driven by the necessity of maintaining a stable ecosystem in the vicinity of the crypt, a critical epithelial regenerative apparatus (49).

In addition, this study shows that this CSCM is broadly conserved in tumoral and normal tissues adjacent to the tumor as wells as in control individuals but is marked by the added presence of pathobionts, i.e., commensal bacteria with pathogenic potential (50), which are highly abundant at the expense of the bona fide CSCM in both tumoral crypts and the associated epithelium. We also found, despite the low number of patients and low abundances, a statistically significantly higher proportion of *S. gallolyticus* (formerly Streptococcus bovis) in tumoral samples than in the adjacent nontumoral samples, in accordance with the results of previous publications (51, 52). We also found an increase in the abundance of B. fragilis in tumoral crypts of right colons, even though this bacterium was also present in normal paired samples. B. fragilis involvement in the oncogenic process leading to CRC (53) was recently confirmed (21, 54). B. fragilis enterotoxin was found to induce reactive oxygen species (ROS)-dependent DNA damage, degradation of the tumor suppressor E-cadherin, and increased expression of Wnt, leading to cell proliferation. Similarly, B. fragilis and *S. gallolyticus* were described as “alpha-bugs,” which by themselves or via microbiota modification can drive the oncogenic process (55). While E. coli strains expressing the polyketide synthase (pks) island, encoding DNA-damaging colibactin (56–58), have also been associated with CRC, this was not confirmed in the present study, as the presence of E. coli was found in only a very low number of patients, without significant differences between tumoral and nontumoral samples. Similarly, we did not find *Providencia* or *Shigella*, two *Proteobacteria* genera previously shown to be associated with the CRC tumor environment (4, 23). In agreement with our results, other studies did not find an increase in E. coli or *Shigella* in cancer groups versus normal patients (3) or a decrease in the abundance of *Escherichia*-*Shigella* (29) or *Shigella* (15) in cancerous versus noncancerous tissues. As a matter of fact, fecal microbiotas of mucosa-associated bacteria significantly differ; hence, fecal samples cannot be considered an adequate reflection of the microbiota attached to mucosal surfaces (24, 40, 41, 59). This needs to be considered before establishing bacterial biomarkers of early stages of CRC.

Interestingly, right-side and left-side tumors appear to differ in their dominant pathobionts, with *Fusobacterium* isolates prevailing in the former and *P. micra* in the latter, both belonging to a decompartmentalized oral microbiota that appears as a hallmark of cancer and inflammatory conditions of the gut (14, 21, 60–64). The association of oral bacteria with tumors is not restricted to colorectal cancer; indeed *P. micra* and *P. stomatis* were found to be enriched in patients with gastric cancer (65). Moreover, some oral pathogens have been associated with a higher risk of pancreatic cancer (66). A “driver-passenger” model was proposed to explain the bacterial interactions occurring in CRC (67). In this model, driver bacteria are involved in the initiation phase of CRC and are then replaced by passenger bacteria that promote tumorigenesis. In other terms, a first change in the gut microbiota allows for oral bacteria to colonize the gut mucosa, and by the disruption of the epithelial barrier, these bacteria promote oncogenesis (60). Many studies mention an association between F. nucleatum and CRC (56). Interestingly, our study identified with the HITdb *F. periodonticum* as associated with CRC. However, by further characterization using *rpoB* sequencing, we confirmed that F. nucleatum subsp. *polymorphum* is actually the species associated with CRC. This result is in line with those of previous phylogenetic studies based on 16S rRNA gene sequence analysis reporting either that *F. periodonticum* is indistinguishable from F. nucleatum (68) or that these two species are very close (69, 70). Nonetheless, *F. periodonticum* has also been previously identified in colon tumors (21, 71). F. nucleatum was proposed as a driver bacterium through its ability to adhere to and invade epithelial cells through its FadA adhesin, followed by an increase in the production of ROS, transcription factors, Wnt, and inflammatory proteins that stimulate the growth of CRC cells (72). Moreover, it was shown that microsatellite instability was present in the right colon but that chromosomal instability was more frequent in the left colon, possibly due to differences in microbiota composition (56, 73).

One limitation of the present and similar studies analyzing low-biomass samples, such as colonic crypt microdissected structures, is the risk of potential environmental contaminations leading to the misinterpretation of a result if one does not apply alternative techniques to 16S rRNA analysis, such as FISH or cultivation, both being impossible to apply to the entire array of 16S rRNA-identified taxa in a complex structure where bacteria are possibly weakly metabolically active. For instance, recent studies on the microbial compositions of placenta and amniotic fluid, which are expected to be poorly, if at all, colonized, indicate that 16S rRNA gene sequencing does not reveal differences in bacterial composition between samples and technical controls (74, 75). As a matter of fact, the main sources of contamination stem from reagents, such as extraction buffers and PCR reagents, or even from cross-contamination between samples. One approach may be to remove the sequences present in negative-control samples. However, low levels of real sequences may be present in the negative controls due to cross-contamination, and the removal of such sequences may result in loss of relevant biological signals (76, 77). Alternatively, one could remove the potential contaminants following 16S rRNA gene sequencing by deleting the sequences previously identified as contaminants in the literature. This might, however, arbitrarily eliminate relevant signals because the sequences were identified as contaminants on the basis of different extraction methods and PCR reagents. Some sequences assigned to phylotypes described in the literature as environmental contaminants, such as Agrobacterium tumefaciens, Pseudomonas monteilii, and *Chryseobacterium*, were detected in our study but not necessarily together in the same samples (Table S1B). Another known environmental contaminant is Acinetobacter. However, using a probe specifically targeting the 16Sr RNA of this genus, we could visualize the presence of Acinetobacter in our colonic human samples, thereby providing the necessary alternative method required to confirm the actual presence of the bacterium. As a reminder, in a previous study bearing on murine colonic crypts, we had been able to confirm the presence of Acinetobacter not only by FISH but also by culture (78). This demonstrates that Acinetobacter is likely to be a universal commensal of colonic crypts following harnessing from environmental sources. In summary, in these studies of low-biomass samples, one is faced with the difficult exercise of extracting relevant signals from among contaminating noise that cannot be rationally eliminated. Confirmation by alternative approaches, such as FISH and culture, brings indisputable validation. The issue is more difficult for taxa that do not benefit from these alternative techniques. One can consider that environmental taxa with high signal levels that share common metabolic properties with Acinetobacter (i.e., strictly aerobic, nonfermentative) may also be considered relevant. One therefore needs to perform a critical analysis on the basis of the above-described criteria but cannot at once delete all signals without reflection.

We are also conscious that a second limitation of the study was the low number of biopsy specimens from healthy volunteers; however, this does not alter the conclusions based on the comparison between tumoral and adjacent nontumoral samples from cancerous patients. In conclusion, based on this decompartmentalization and the differential microbial patterns present in the distinctive colon segments, the role of these pathobionts, such as *Fusobacterium* and Bacteroides fragilis, awaits further evaluation, as they may reveal key elements on the mechanisms involved in colon oncogenesis.

## MATERIALS AND METHODS

### Patient characteristics.

A total of 58 patients (Table 1) underwent surgical resection of primary colonic adenocarcinoma at the Hôpital Henri Mondor, Créteil, France. Following resection, colonic specimens were examined by an oncological pathologist, and biopsy specimens were taken from the tumor site and from the adjacent nonmalignant tissue (at a distance of about 15 to 20 cm). In addition, nine colonic biopsy specimens from routine colonoscopy procedures (patients S1 to S9) were included in the analysis and served as normal tissue controls (Table 1). The right and left colon surgical biopsy specimens were obtained without prior cleansing, while for the rectal samples and those collected during colonoscopies, patients were prepped using two liters of polyethylene glycol the day prior to the procedure. This cleansing treatment eliminates the bulk of luminal microbes, and as a consequence, the entry of bacteria into the crypts is very inefficient. A small piece of each tissue was immediately snap-frozen in nitrogen and subsequently embedded into optimal cutting temperature (OCT) compound 4583 (Sakura), subjected to dry ice-chilled isopentane, and stored at −80°C.

### LCM and sample collection for intestinal microbiota analysis.

Frozen blocks were cut at a thickness of 8 μm using a CM 3050S cryostat (Leica), and sections were collected on Arcturus PEN membrane glass slides (Biosystems) and stored at −20°C until use. Frozen sections were thawed and briefly stained with histogen (MDS Analytical Technologies) containing RNaseOUT recombinant RNase inhibitor, washed in RNase-free water supplemented with ProtectRNA (Sigma-Aldrich), and dehydrated in ethanol (once in 70% [vol/vol] ethanol for 30 s, twice in 95% [vol/vol] ethanol for 1 min, and twice in 100% [vol/vol] ethanol for 2 min) and in xylene (two incubation rounds of 5 min) before being air-dried. Slides were then transferred into a Veritas LCM system (Arcturus XT microdissection system; ThermoFisher Scientific), microdissected, and captured on Capture HS LCM caps (Arcturus; ThermoFisher Scientific). For each sample, crypts and mucosa-associated regions were microdissected. DNA was extracted using the PicoPure DNA extraction kit (Arcturus; ThermoFisher Scientific) after incubation for 30 min at room temperature with lysozyme (10 mg/ml in phosphate-buffered saline [PBS]; Sigma-Aldrich). Negative controls included extractions without addition of any sample to evaluate potential contamination from kit reagents. To minimize further risk of contamination from small materials, plastic tubes and plates were pretreated with UV cross-linker for 3 h before use.

### 16S rRNA gene sequencing and analysis.

16S rRNA gene amplification and library construction were performed according to Illumina recommendations (79). Briefly, a first PCR round was performed using 4 μl of DNA extracted from microdissected tissues using primers targeting the 16S rRNA gene V3 and V4 regions, forward primer 341F (5′-*TCGTCGGCAGCGTCAGATGTGTATAAGAGACAGCC*TACGGGNGGCWGCAG-3′), and reverse primer 805R (5′*-GTCTCGTGGGCTCGGAGATGTGTATAAGAGACAG*GACTACHVGGGTATCTAATCC-3′), where the Illumina adapters are indicated in italics. After 25 cycles, PCR products were purified using AMPure XP beads (Beckman Coulter Genomics) according to the manufacturer’s recommendations. A second PCR was performed to attach dual indices using the Nextera XT index kit (Illumina). After eight cycles, PCR products were purified using AMPure XP beads (Beckman Coulter Genomics). PCRs were also performed using PicoPure extraction buffer alone (DNA extraction negative controls) or water as the template and used as controls for 16S rRNA gene sequencing analysis. The size of the libraries (∼600 bp) and their quantification were determined by Fragment Analyzer (Advanced Analytical) using the high-sensitivity NGS fragment analysis kit. Purified amplicons were pooled in equimolar concentrations to obtain a 6 pM library containing 10% of the PhiX control. Sequencing was performed on an Illumina MiSeq instrument using the paired-end 300-bp protocol at the Institut Pasteur.

We sequenced a total of 269 samples, including 228 laser-microdissected samples, 22 PicoPure buffer controls (LCM buffer), and 19 water samples used as the template controls. A total of 41,939,989 read pairs were generated (mean, 155,911 read pairs per sample, with a median of 87,190). The reads were first demultiplexed according to their dual barcode by sample, and each pair was assembled using FLASH v1.2.11 (80). The FLASH error correction feature was used to remove ambiguous base pairs in the overlapping region of each read pairs (the average size of the overlapping region was 120 bp). Primer sequences were then removed (cutadapt 2.6) (81), and any sequences containing ambiguous bases (N) or fewer than 370 bases (PRINSEQ-lite 0.20.3) were removed (82). Furthermore, only fragments with a mean Phred quality score above 28 were kept. After removal of all buffer control and water samples, a total of 11,005,482 sequences were retained, with an average of 48,269 sequences per sample (median 20,127). Sequences analyses were performed using QIIME (v1.9.1) (83) as follows: (i) pick_otu.py was used to cluster sequences using the UCLUST algorithm (84) into OTUs at 97% similarity; (ii) pick_rep_set.py was used to select a representatives set of OTUs, including a sequence representative of each OTU, corresponding to the centroid of the associated cluster; and (iii) assigne_taxonomy.py was used to perform taxonomy assignment for the representative set, using the UCLUST assignment method (default parameters) and the Greengenes 13.8 16S rRNA gene sequence database (35) or the human intestinal 16S rRNA gene taxonomic database, HITdb (36). To build the phylogenic tree of representative sets, the multiple alignment was generated using PyNAST (85) based on default similarity of 75%. The phylogenic tree was constructed using make_phylogeny.py with the tree_method_default parameter using the FastTree algorithm (86). The resulting phylogenic tree was further processed to calculate core diversity metrics, including β-diversity (based on weighted/unweighted UniFrac metrics [87]) and α-diversity (for different depths of rarefaction) measures. Moreover, we carried out taxonomic group diversity analysis and differential taxonomy abundance (scripts of QIIME). The intergroup high similarity and intragroup low similarity of microbiotas are assessed by determining β-diversity using PCoA (generated by QIIME using unweighted UniFrac metrics) (83, 87). To test for significant differences in taxonomic abundances, we used the nonparametric Kruskal-Wallis test with the false-discovery rate correction implemented in QIIME and the zero-inflated Gaussian (ZIG) mixture model (42). Differences were considered significant at a *P* of *<*0.05. The number of sequences obtained with the control samples was not sufficient to obtain significant results.

The core OTU was defined as the set of OTUs that were observed in a given fraction of samples. Before computing the core OTU, all numbers of OTU less than 5 were considered 0. A fraction of 50% was used, and the core OTU was calculated using compute_core_microbiom.py.

### Quantitative RT-PCR.

Diluted DNAs extracted from the LCM crypt or luminal part of the colon were used as a template for quantitative reverse transcription (RT)-PCR using specific primers (400 nM) for phyla and/or bacterial families in a 15-μl final volume containing Sybr green master mix (Roche) using the QuantStudio 7 flex real-time PCR system (Applied Biosystems). Cycling conditions were as follows: an initial denaturation step at 95°C for 10 min, with 40 cycles of denaturation at 95°C for 10 s, and an annealing/elongation at 60°C for 60 s. The list of all primers used in this study is provided in Table S4 in the supplemental material. The specificity of the primers was tested using dilution series of genomic DNA from Escherichia coli prepared by using the Wizard kit from Promega and using genomic DNAs of *Fusobacterium*, Bacteroides fragilis, and Parvimonas micra obtained from the DSMZ. Dilutions of bacterial genomic DNA were performed in water or with an extract of human colonic tissue.

10.1128/mBio.01315-19.10 TABLE S4 Primer and probe sequences used in this study, with their respective references. Download Table S4, DOCX file, 0.1 MB. Copyright © 2019 Saffarian et al. 2019 Saffarian et al. This content is distributed under the terms of the Creative Commons Attribution 4.0 International license.

### PCR amplification of the *Fusobacterium rpoB* gene.

A total of 2 μl of the DNA extracted from LCM crypts was used as the template in a final volume of 50 μl with 1 U of *Taq* DNA polymerase (MP Biomedicals). The PCR conditions for amplifying the *rpoB* gene were as follows: denaturation at 94°C for 1 min, primer annealing at 50°C for 1 min, and extension at 72°C for 1 min. The final cycle included an additional extension time of 10 min at 72°C. A 2-μl aliquot of the reaction mixture was analyzed by 1.5% agarose gel electrophoresis in a Tris-acetate buffer at 100 V for 45 min. The amplification products were stained with ethidium bromide and visualized by UV transillumination. The PCR products were purified using the QIAquick PCR purification kit (Qiagen). The nucleotide sequences were determined by Sanger sequencing (Eurofins Genomics).

### FISH.

Frozen sections were rehydrated in PBS, incubated with 4% paraformaldehyde, washed twice in PBS, covered with a solution of lysozyme at 10 mg/ml in PBS for 10 min at 37°C, and washed twice with PBS. After 30 min of incubation in hybridization buffer (20 mM Tris-HCl [pH 8], 0.9 M NaCl, 0.01% SDS, 30% formamide), slides were incubated overnight in hybridization buffer containing a 50 nM or 100 nM concentration of the fluorescent probes at 55°C (Table S4). After being washed twice in 64 mM NaCl, 24 mM Tris-HCl, 5 mM EDTA 0.1% SDS, slides were covered for 30 s with 4′,6-diamidino-2-phenylindole (DAPI) (0.125 μg/ml in PBS), washed in PBS, and mounted in ProLong gold antifade reagent (Invitrogen). The fluorescent 16S rRNA-targeted oligonucleotide probes used in this study are listed in Table S4 in the supplemental material. The probes were covalently linked to Alexa 555 or Alexa 488 at their 5′ ends. The labeled nonEub338 probe was used as control, and no staining was observed. Slides were examined under an Olympus IX81 microscope equipped with a charge-coupled device (CCD) camera, and images were processed using the MetaVue software program or under a Widefield ApoTome inverted microscope (Zeiss) using the AxoVision software program. FISH images proposed for this paper did not allow us to spot bacteria inside all the colonic crypts. Indeed, FISH images were acquired with a single-focus plan, and when the focus plan changed, bacteria disappeared in previously stained crypts and appeared in other crypts due to uneven localization of crypts in the blocks and possibly different localizations of the relevant bacteria in the height of the crypts (Fig. S6). Moreover, when crypts that contain bacteria are visualized and counted after FISH by varying the focus plan, bacteria are present in 70% of colonic crypts.

10.1128/mBio.01315-19.6 FIG S6 Representative pictures from FISH experiments at two different foci. FISH analyses with the pan-bacterial probe Eub338 (red) of nontumoral human colonic tissue using two different focus plans (left and right). Nuclei are counterstained with DAPI (green). Download FIG S6, JPG file, 0.7 MB. Copyright © 2019 Saffarian et al. 2019 Saffarian et al. This content is distributed under the terms of the Creative Commons Attribution 4.0 International license.

### Data deposition.

The 16S rRNA gene sequence data have been deposited into the NCBI Sequence Read Archive database under BioProject accession number PRJNA507548.
