# Supplementary figures and images for: Crypt- and Mucosa-Associated Core Microbiotas in Humans and Their Alteration in Colon Cancer Patients
Source: mBio. 2019 Jul 16;10(4):e01315-19. doi: 10.1128/mBio.01315-19 (PMC6635529; doi:10.1128/mBio.01315-19)

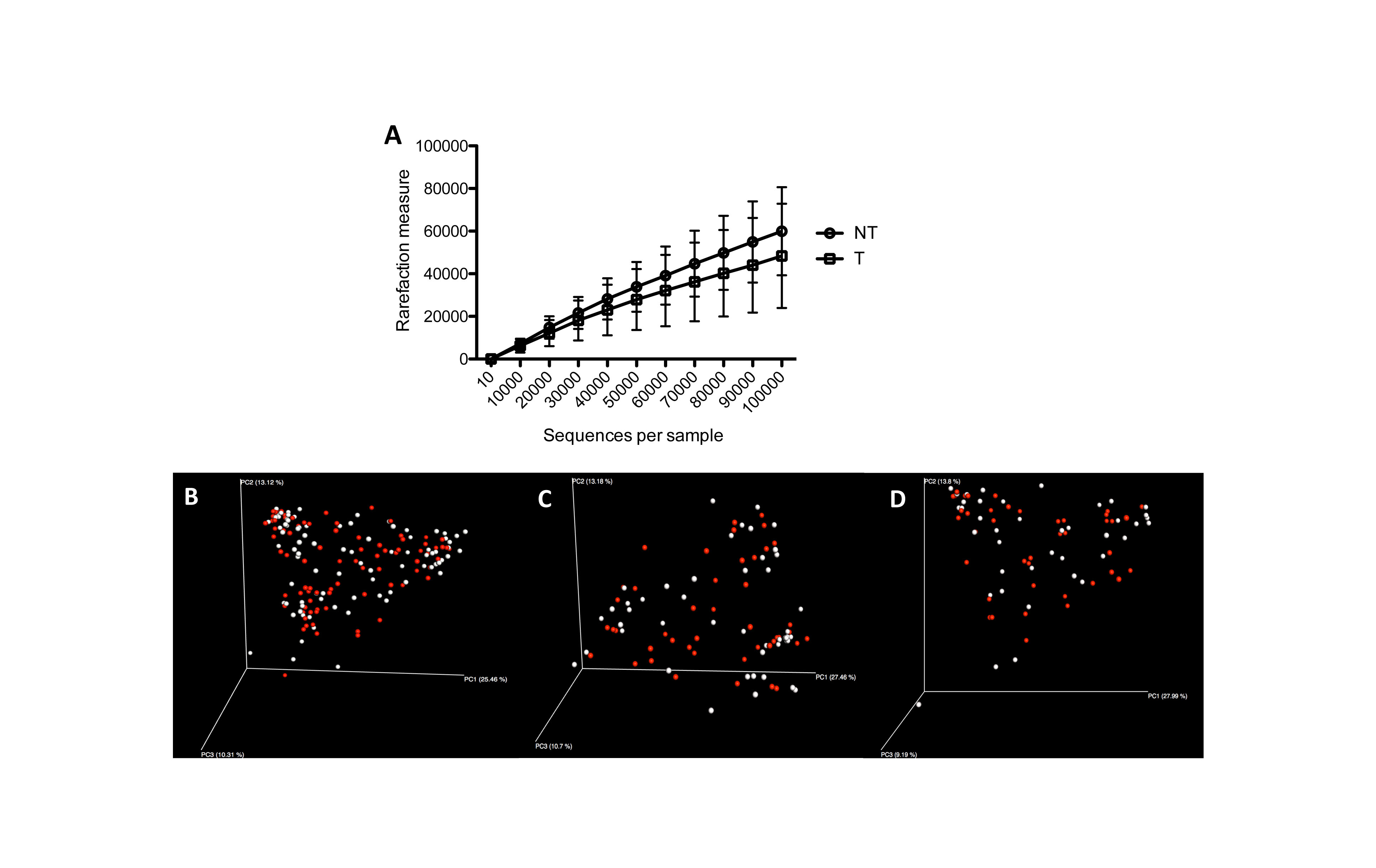

Supplement: FIG S1 [file mBio.01315-19-sf001.jpg]

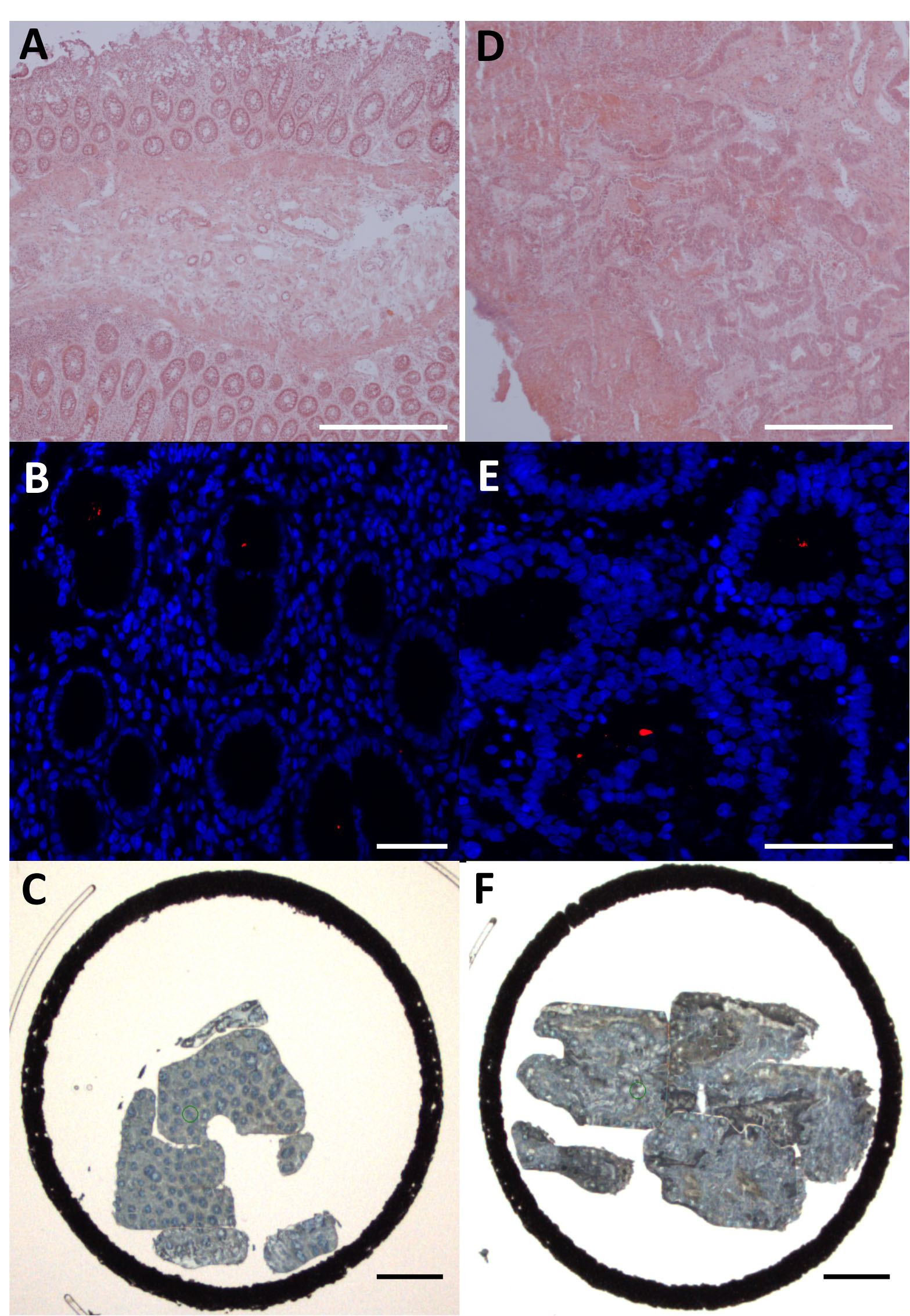

Supplement: FIG S2 [file mBio.01315-19-sf002.jpg]

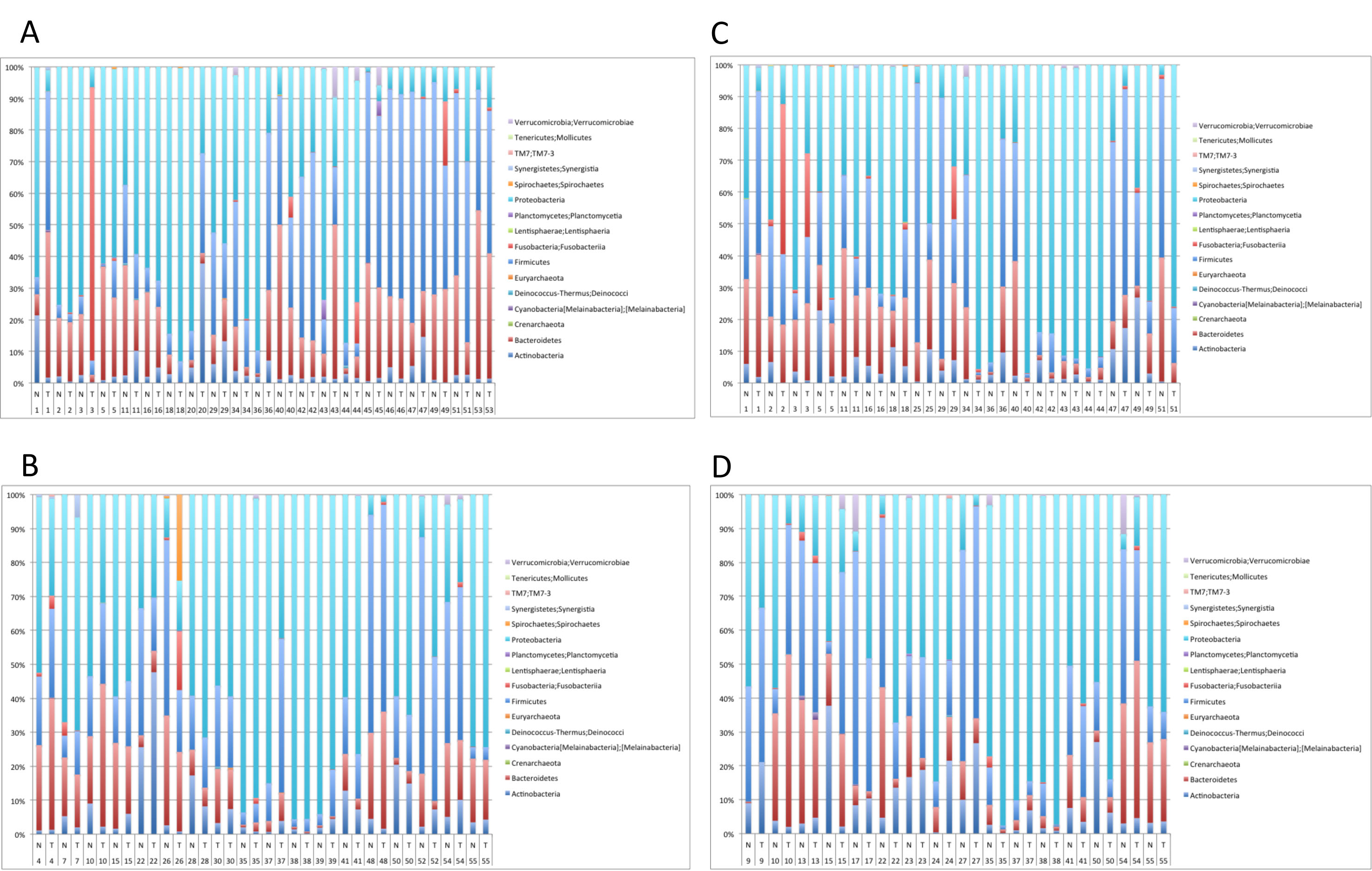

Supplement: FIG S3 [file mBio.01315-19-sf003.jpg]

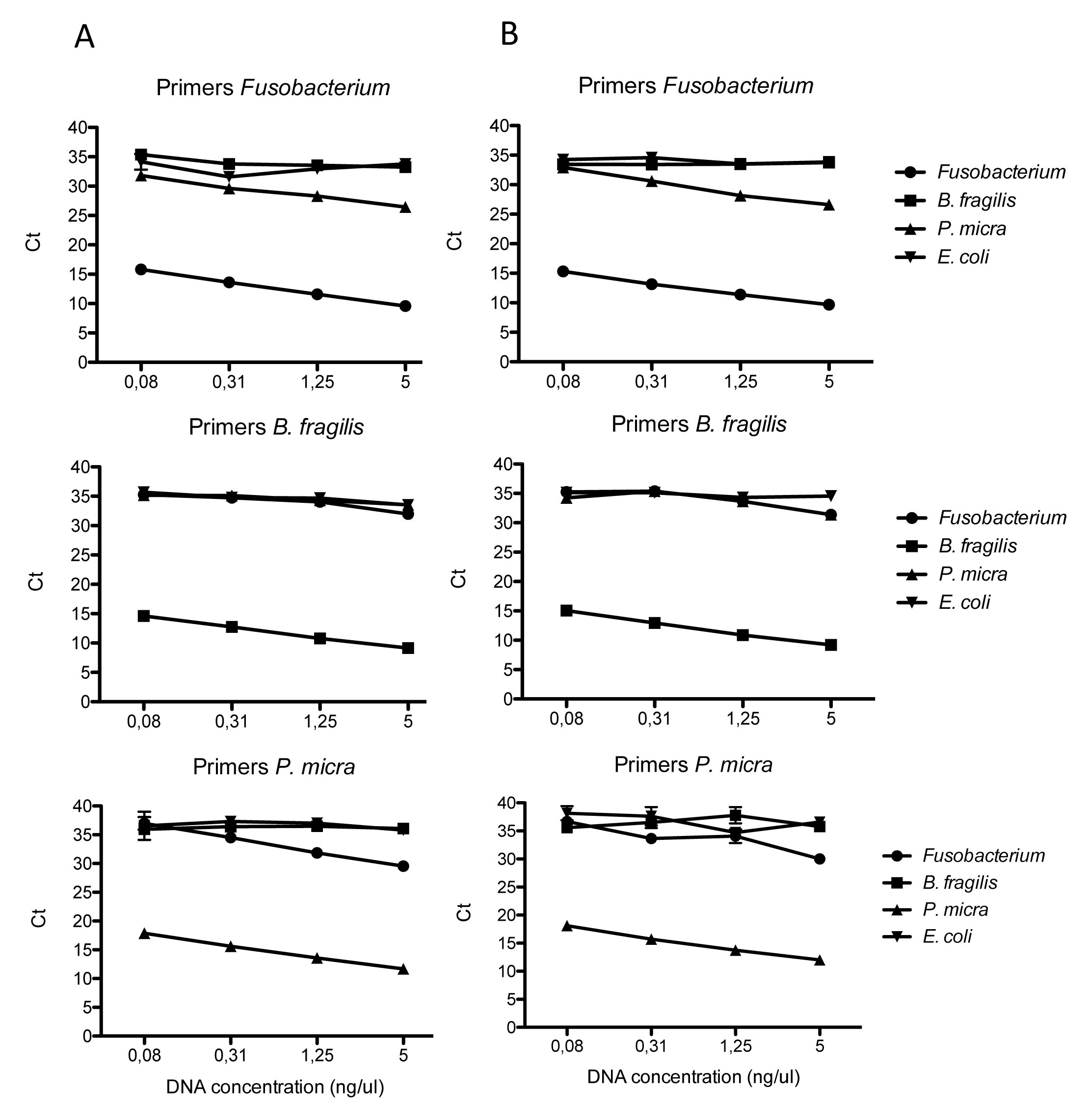

Supplement: FIG S4 [file mBio.01315-19-sf004.jpg]

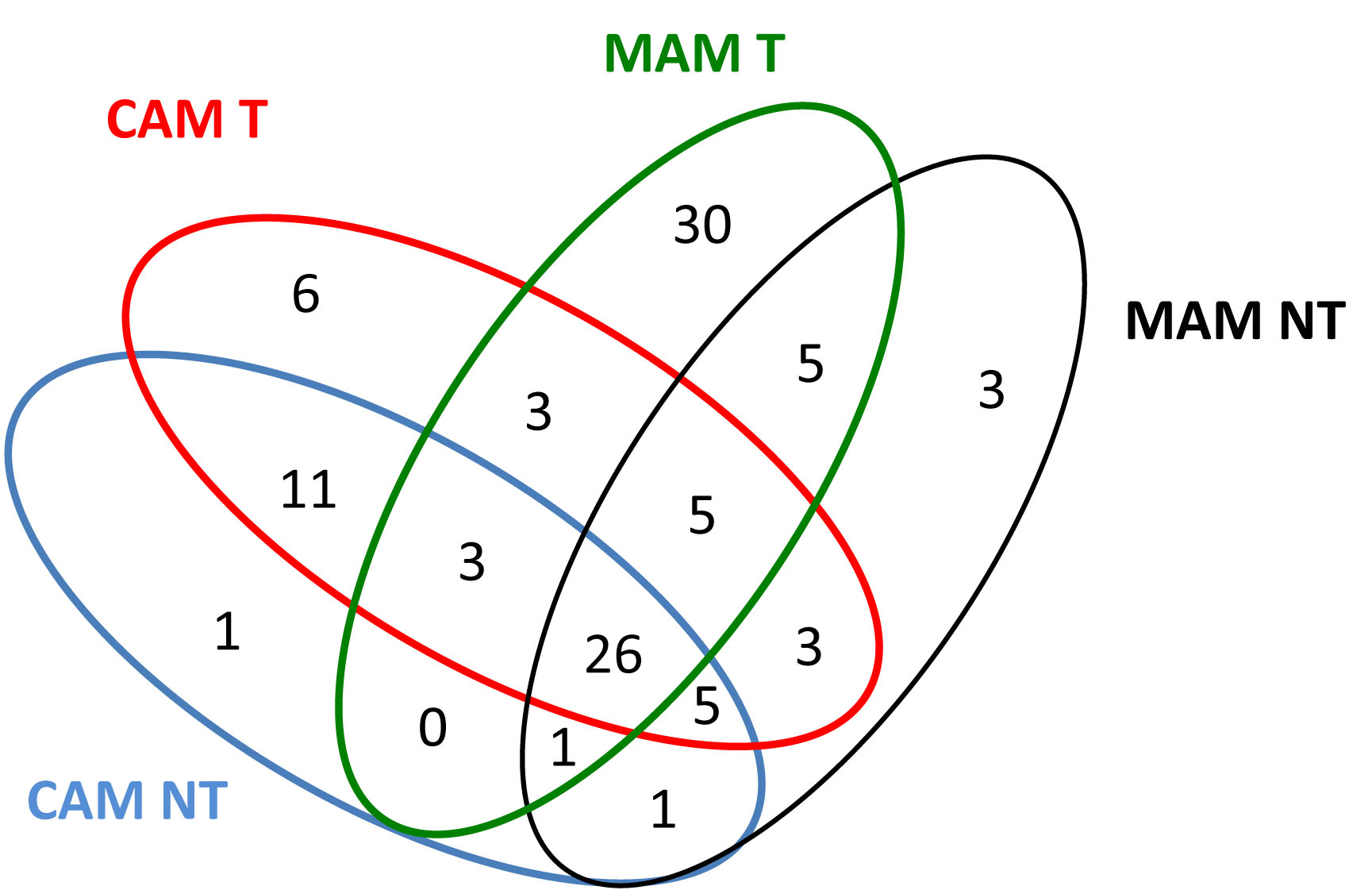

Supplement: FIG S5 [file mBio.01315-19-sf005.jpg]

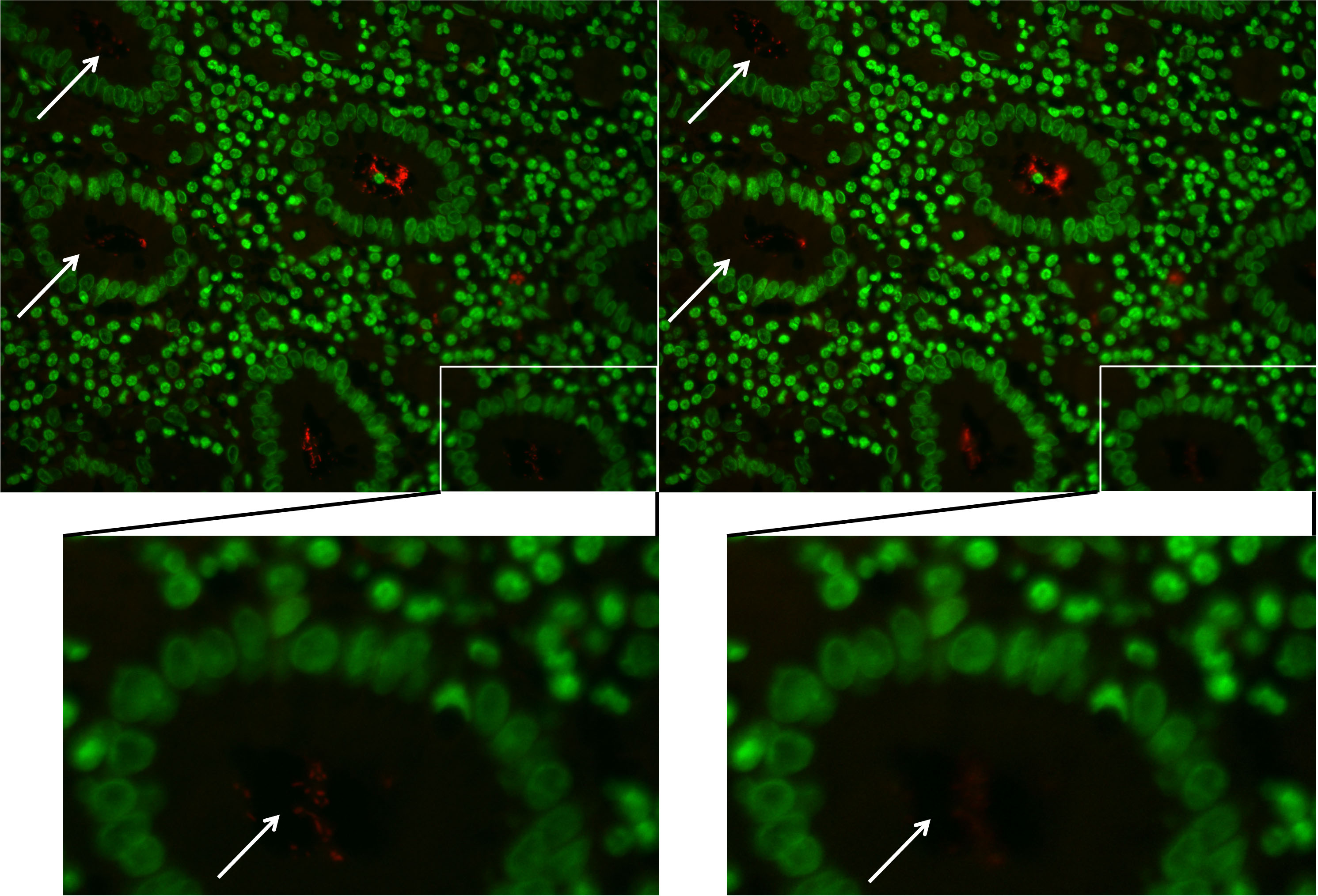

Supplement: FIG S6 [file mBio.01315-19-sf006.jpg]
