# Supplementary material for: Crypt- and Mucosa-Associated Core Microbiotas in Humans and Their Alteration in Colon Cancer Patients
Source: mBio. 2019 Jul 16;10(4):e01315-19. doi: 10.1128/mBio.01315-19 (PMC6635529; doi:10.1128/mBio.01315-19)
Supplement: TABLE S4 [file mBio.01315-19-st004.docx]

**Probes used for FISH**

| Probe | Sequence (5’-3’) | Formamide/Temperature | Concentration | References |
| --- | --- | --- | --- | --- |
| Eub338 | GCTGCCTCCCGTAGGAGT | 30 % / 55°C | 50 nM | Amann RI et al. |
| nonEub338 | ACTCCTACGGGAGGCAGC | 30 % / 55°C | 50 nM | Amann RI et al. |
| *Fusobacterium* (FUS664) | CTTGTAGTTCCGCYTACCTC | 40 % / 55°C | 100 nM | Greuter D et al. |
| *B. fragilis* (Bfrag_998) | GTTTCCACATCATTCCACTG | 30 % / 35°C | 50 nM | Greuter D et al. |
| *Acinetobacter* (ACA652) | atcctctcccatactcta | 30 % / 55°C | 50 nM | Wagner M et al. |

**Primers used for RT-PCR**

| Target | Forward (5’-3’) | Reverse (5’-3’) | References |
| --- | --- | --- | --- |
| All Bacteria  (16SrDNA) | F_Bact1369  CGGTGAATACGTTCCCGG | R_Prok1492  TACGGCTACCTTGTTACGACTT | Suzuki MT et al. |
| *Fusobacterium* | GGATTTATTGGGCGTAAAGC | GGCATTCCTACAAATATCTACGAA | Burns MB et al. |
| *Bacteroides fragilis* | TCRGGAAGAAAGCTTGCT | CATCCTTTACCGGAATCCT | Tong J et al. |
| *Parvimonas micra* | AACGACGATTAATACCGCATGAGACC | CTTCCTCCTATGATACCGTCATTA | Eick S et al. |
| *B. fragilis* toxin (bft368) | GAACCTAAAACGGTATATGT | GTTGTAGACATCCCACTGGC | 54 |
| Fn-RpoB-1 | CTKGATGAAGAAACAGGAGART | AGTAGCAAGYGAYCCAATAAGT | 67 |
| Fn-RpoB-2 | AACACCAGAAGGACCAAAYATT | ATATCYCCYGGTCCTACTTCT G | 67 |

**Supplemental References**

Amann RI, Binder BJ, Olson RJ, Chisholm SW, Devereux R, Stahl DA. 1990. Combination of 16S rRNA-targeted oligonucleotide probes with flow cytometry for analyzing mixed microbial populations. Appl Environ Microbiol. 56(6):1919-1925.

Burns MB, Lynch J, Starr TK, Knights D, Blekhman R. 2015. Virulence genes are a signature of the microbiome in the colorectal tumor microenvironment. Genome Med. 7(1):55. doi: 10.1186/s13073-015-0177-8.

Eick S, Straube A, Guentsch A, Pfister W, Jentsch H. 2011. Comparison of real-time polymerase chain reaction and DNA-strip technology in microbiological evaluation of periodontitis treatment. Diagn Microbiol Infect Dis. 69(1):12-20. doi: 10.1016/j.diagmicrobio.2010.08.017.

Greuter D, Loy A, Horn M, Rattei T. 2016. probeBase—an online resource for rRNA-targeted oligonucleotide probes and primers: new features. Nucleic Acids Res. 10.1093/nar/gkv1232.

Suzuki MT, Taylor LT & DeLong EF. 2000. Quantitative analysis of small-subunit rRNA genes in mixed microbial populations via 5-nuclease assays. Appl Environ Microbiol. 66, 4605–4614.

Tong J, Liu C, Summanen P, Xu H, Finegold SM. 2011. Application of quantitative real-time PCR for rapid identification of Bacteroides fragilis group and related organisms in human wound samples. Anaerobe. 17(2):64-8. doi: 10.1016/j.anaerobe.2011.03.004.

Wagner M, Erhart R, Manz W, Amann R, Lemmer H, Wedi D, Schleifer KH. 1994. Development of an rRNA-targeted oligonucleotide probe specific for the genus Acinetobacter and its application for in situ monitoring in activated sludge. Appl Environ Microbiol. 60(3):792-800.
